# The comparative effects of exercise type on motor function of patients with Parkinson’s disease: A three-arm randomized trial

**DOI:** 10.3389/fnhum.2022.1033289

**Published:** 2022-12-01

**Authors:** Fang Li, Dongyu Wang, Xiaohong Ba, Zhan Liu, Meiqi Zhang

**Affiliations:** ^1^Department of Neurology, The First Affiliated Hospital of Jinzhou Medical University, Jinzhou, China; ^2^Department of Neurology, The Center Hospital of Jinzhou, Jinzhou, China; ^3^Department of Physical Education and Health Education, Springfield College, Springfield, MA, United States; ^4^Learning-Based Recovery Center, Yale University, New Haven, CT, United States

**Keywords:** exercise, dance, rhythmic auditory stimulation, Parkinson’s disease, motor symptom

## Abstract

**Background:**

Yang-ge dancing is a culturally specific exercise in which people are required to perform motor skills in coordination with rhythmic music. As an integrated exercise with both physical (decelerating the progression of aged-related motor function degeneration) and mental benefits, it has gained great popularity in China, especially among middle-aged and older adults. It remains largely unknown whether Yang-ge dancing (YG) can effectively improve main symptoms of Parkinson’s disease (PD), while conventional exercise rehabilitation program has been recommended in the hospital setting. To this end, this study aimed to investigate the comparative effects of exercise therapy on motor function of PD patients.

**Materials and methods:**

A sample of 51 PD patients were randomly assigned to Yang-ge dancing, conventional exercise, or conventional exercise with music. Participants in each group performed 60 min per session, five sessions per week of interventions for 4 weeks. All the participants were assessed using the Unified Parkinson’s Disease Rating Scale—motor examination, Berg balance test, timed up and go test, and Purdue pegboard test. Motor performances were examined before and after intervention.

**Results:**

All the three groups were benefited from exercise. Compared to conventional exercise, the Yang-ge dancing and conventional exercise with music had additional positive effects in mobility with reference to baseline.

In addition, compared to the two conventional exercise groups (either with/without music), the Yang-ge dancing further enhanced manual dexterity.

**Conclusion:**

Exercise with rhythmic auditory stimulation optimized mobility in PD, while YG dance specifically contributed to improvement in manual dexterity.

**Clinical trial registeration:**

[https://clinicaltrials.gov/], identifier [ChiCTR2200061252].

## Introduction

Motor dysfunction adversely affect activities of daily living of patients with Parkinson’s disease (PD), which require a great attention (Li et al., 2012). Since motor function is less responsive to pharmacological treatments (Li et al., 2012), researchers have recognized exercise as an alternative strategy and carried out many forms of exercise intervention trials (Petzinger et al., 2013; Baek et al., 2020; De la Rosa et al., 2020; Jung et al., 2020; Ezzatvar et al., 2021; Liu et al., 2021). Specifically, based on posttest, general motor function (reflected by the Unified Parkinson’s Disease Rating Scale—Motor Examination, UPDRS—Motor) significantly improved in PD patients who were treated with exercise therapy (Ni et al., 2016; Kwok et al., 2019). In addition, the effects of exercise therapies on balance and mobility have been most closely examined, whereby patients with PD who received exercise treatment demonstrated significant improvements in postural control and walking ability (Zhang et al., 2015; Shanahan et al., 2017). However, as an equally important health indicator, manual dexterity of PD patients has not received enough attention in previous exercise interventional studies. Against this background, researchers have recently investigated the impacts of exercise on hand dexterity, but results are still inconsistent (Mateos-Toset et al., 2016; Allen et al., 2017; Kalyani et al., 2020). Additionally, it still remain largely unknown whether a culturally specific exercise modality is beneficial for the above-presented measures of motor function of PD patients as the conventional therapy is more acceptable in both eastern and western society.

Yang-ge is a representative form of Han Chinese folk dance. Yang-ge is originated from farming activities, in which farmers pray for or celebrate the harvest, and was gradually developed into a dance form for health promotion of Chinese people. The Yang-ge dance featured in cheerful tones and brisk movement. Yang-ge is assumed to be beneficial to PD patients as it mimics the major characteristics of current exercise modalities that have been examined to be beneficial: Sufficient lower-limb practice and exercise with rhythm (van Eijkeren et al., 2008; Mateos-Toset et al., 2016; Calabrò et al., 2019). Additionally, Yang-ge particularly highlights hand movements where participants practice with holding light props (e.g., hand fan and handkerchief), to complement the conventional exercise forms. In the present study, the well-documented rhythmic auditory stimulation (RAS) was also used for improving motor-related function as it is a technique of neurologic music therapy that synchronizes gait movements to predictable time cues (Thaut et al., 1996; McIntosh et al., 1997). Integrating RAS into PD exercise protocols has been shown to help the improvement of gait and balance performance (Kadivar et al., 2011; Suzuki et al., 2019). This can be attributed to the excitability of spinal motor neurons is increased by the RAS patterns (Suzuki et al., 2019).

Taken together, the current study aimed to (a) investigate the effects of exercise therapy and RAS and (b) examine the feasibility of Yang-ge dancing (YG) for PD. The substantive hypotheses were that Yang-ge dancing is an effective and feasible strategy to improve motor function of patients with PD, and patients in the Yang-ge group would have better improvements in motor function compared to the conventional exercise groups either with or without RAS.

## Materials and methods

The study featured a three-arm randomized controlled research design. The independent variables were treatment [Yang-ge dancing (YG), conventional exercise (CE), and conventional exercise plus music (CEM)] and time (baseline and posttest). The dependent variables included behavioral testing data and adverse-events records. Participants, measurements, procedures, and data analyses are discussed in this section.

### Participants

An *a priori* power analysis was conducted using G*Power (Faul et al., 2007) to determine the minimum sample size required for the present study. Results indicated that to achieve 80% power for detecting a medium effect, at a significance criterion of α = 0.05, was *N* = 45. Thus, the obtained sample size of *N* = 55 was adequate enough to test the study hypothesis. The participants were recruited from two hospitals in northeastern China by means of media advertisements, and referrals from neurologists or physical therapists.

Eligibility criteria include: (1) a clinical diagnosis of PD with a disease severity rating of stage 1–3 on the H&Y scale; (2) stable medication use; (3) ability to walk unaided and control objects (e.g., holding a mug); (4) medical clearance for participation; (5) no obvious cognitive or perceptual impairment (able to follow instructions during assessment and intervention); (6) willingness to be assigned to any one of the groups; and (7) currently not be involved in any other experimental program(s). Exclusion criteria include: (1) not aged from 40 to 85 years; (2) unstable medication use; (3) unable to walk unaided and control objects; (4) physician does not approve or recommend participation; and (5) did not return the informed consent form.

### Procedures

**FIGURE 1:**
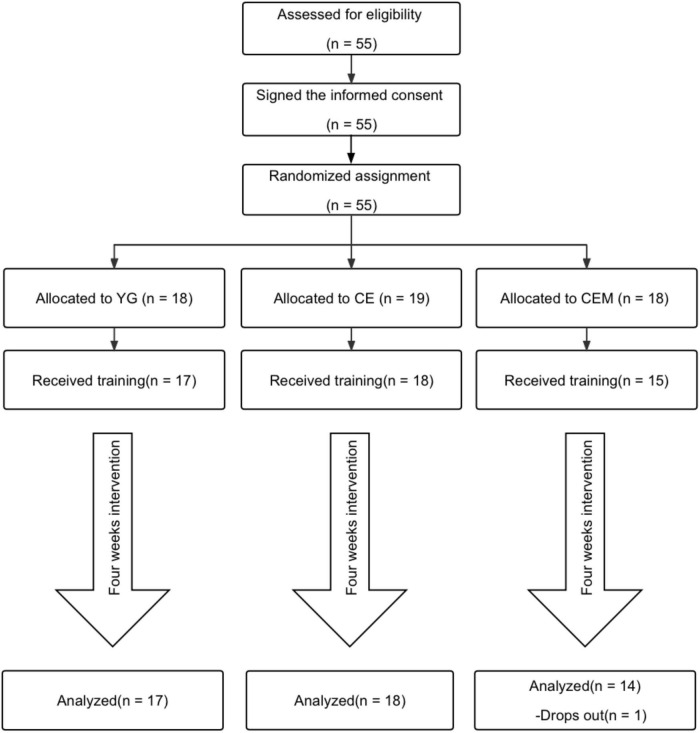
Flowchart.

Approval of methods and procedures was obtained from the Institutional Review Board (IRB) of a college in western Massachusetts, U.S. After receiving approval from the Institutional Review Board, patients who volunteered and met the eligibility criteria for the study attended the assessments and exercise treatments. The whole process is shown in Figure 1.

During the intervention, participants in each group performed 60 min per session, five sessions per week of interventions for 4 weeks. At least three sessions per week were required, but participants were encouraged to attend five sessions weekly. The baseline and post-intervention assessment were conducted in 1 week before and after the 4-week intervention. All the patients were required to not consume any alcohol, caffeine, or tobacco for at least 10 h before the assessments (Silveira et al., 2010).

### Intervention strategies

Participants were randomly assigned to one of three intervention groups (i.e., YG, CE, or CEM) and received the relevant intervention. The purpose of integrating two active control groups was to counteract the confounding effects of exercise modalities and RAS. The protocols ensured that each group had a consistent intervention schedule on duration (i.e., 60 min per session), frequency (i.e., five times per week), and training structure (i.e., a 5–10-min warm-up, a 45–50-min core activity session, and a 5-min cooldown).

In addition, for security concerns, each protocol covered at least half seated positions practice, and two research assistants or physicians monitored the whole treatments. The participants were provided a general safety precautions checklist before the treatment started (Best-Martini and Jones-DiGenova, 2014).

#### Yang-ge dancing

The Yang-ge dancing practice started with the learning of movement elements, and then gradually transitioned to the practice of dancing routines. Over the practice, each participant was instructed to dance with holding a light prop in each hand (i.e., a hand fan and a towel). The *Primary Tutorial of Chinese Folk Dance by Beijing Dance Academy* (Jia and Zhong, 2004) was adopted to develop the Yang-ge exercise protocols. The practice tasks are presented in Supplementary Table 1. All the learning and practice were performed with music.

#### Conventional exercise

Participants who were allocated to the CE group were treated with conventional PD exercise protocols. The exercise protocols were developed by referring to the prescriptions provided by the Parkinson’s Foundation and *Exercise for frail elders, Second edition* (Best-Martini and Jones-DiGenova, 2014). The exercise protocols covered flexibility, balance, strength, cardio, and coordination training. A list of practice tasks is presented in Supplementary Table 2. No background music or auditory cues were provided during the exercise treatment. Instead, the instructor led the practice by counting the repetitions or clocking the time of maintaining a posture.

#### Conventional exercise plus music

Participants in the CEM group performed the same exercise prescriptions with the CE but the whole exercise process was implemented with music-based rhythmic auditory stimulation. The music pace to flexibility, strength, and balance training was relatively mild, whereas the speed for coordination and cardio exercise was fast.

### Measurements

Variables that were measured in this study include UPDRS—motor examination, Berg balance test (BBS), timed up and go test (TUG), and Purdue pegboard test (PPT). The baseline and post-intervention assessments were conducted in 1 week before and after the exercise treatment. To reduce the potential impacts of time and medication, all the examinations were conducted in the mornings in a week before and after the intervention. All the measurements were done by physicians who were blinded to group assignment.

#### UPDRS—Motor examination

The UPDRS—motor examination consists of 18 movement assessments (e.g., speech, facial expression, rigidity, hand movement, gait, and posture; Goetz et al., 2008). Based on the degree of impairment, each item scores from 0 to 4, a 0 indicates no symptom or normal function, a 4 suggests severe impairment.

#### Berg balance test

The BBS contains 14 items that could be used to quantitatively assess balance and risk for falls in older adults through direct observation of performance (Blum and Korner-Bitensky, 2008). Each participant was guided to complete the following tasks: sitting to standing, standing unsupported, sitting unsupported, standing to sit, transfers, standing with eyes closed, standing with feet together, reaching forward with an outstretched arm, retrieving an object from the floor, turning to look behind, turning 360 degrees, placing an alternate foot on the stool, standing with one foot in front, and standing on one foot (Berg et al., 1995). The items are scaled from 0 to 4, with a score of 0 being unable to complete the task and a score of 4 being able to complete the task independently. The complete score is calculated out of 56 possible points. Scores under 20 represent balance dysfunction, between 21 and 40 represent acceptable balance ability, and above 41 represent normal balance ability. A chair, a stopwatch, a ruler, and a step are required as equipment to conduct the assessment.

#### Timed up and go test

Each participant was instructed to complete the following tasks: stand up from a chair, walk for 3 meters, turn, walk back to the chair, and sit down again (Podsiadlo and Richardson, 1991). The test administrator measures the time taken (in seconds) by the process. Participants were allowed to use a stick, but no physical assistance from others was allowed during the process. A stopwatch, a standard armchair, a cone, and a tapeline are required for the test.

#### Purdue pegboard test

The PPT testing material is composed of a board with pins, collars, and washers (located in cups at the top of the board). The board contains two parallel rows with 25 holes on each side. Each participant was required to sequentially complete the tasks by right hand, left hand, both hands simultaneously, and assembly. In the first three tests, the participant places the maximum number of pins within 30 s. In the assembly test, the participant used alternate hands to make assemblies consisting of pins, collars, and washers within 60 s. The tester counted the numbers of pins inserted in the holes (for the first two tests), the pairs of pins (for the both-hands test), and the number of pins, collar, and washers assembled (for the assembly test).

#### Feasibility

Within the exercise programs, the safety and feasibility outcomes were assessed. Adherence indicators include the drop-out rates (%), missing sessions (%), and adverse events (i.e., fall, muscle soreness/pain, dizziness/faintness, symptom of hypotension, sprain, and low back pain) were documented throughout the trial.

### Data analysis

Between group differences in demographic data and adverse-events frequency were tested with chi-square test (categorical variables) and one-way analysis of variance (ANOVA, continuous variables). Categorized variables were described by frequencies. Continuous variables were presented as mean ± standard deviation (SD).

The 2 × 3 (time × group) mixed multivariate analysis of variance (MANOVA) with the repeated measures was performed to detect the intervention effects on the outcome measures. The independent variables were testing occasions (baseline and posttest) and interventions (YG, CE, and CEM). The dependent variables included UPDRS—motor section, Berg balance test, TUG, and PPT. Data normality, outliers, and homogeneity of variance were checked before conducting statistical analyses. The Z score criterion was used as the whole sample outlier criterion, which a value out of ±3.29Z is defined as an outlier. Box plot was used to detect the within-group outliers. Considering the limited sample size, to save the information maximally, the winsorizing method was applied for potential outlier(s). Histograms and skewness/kurtosis ratios were used to check the normality. Factorial ANOVAs with repeated measures were applied as the *post-hoc* analyses; simple effect tests were then used for exploring the specific group differences.

Demographic and behavioral data were analyzed using SPSS version 23.0 for Mac (IBM Corp, NY, USA) and graphed by GraphPad Prism version 8.0.0 for Mac (GraphPad Software, CA, USA). The alpha level for statistical procedures was set at 0.05.

## Results

### Baseline characteristics

A total of 55 patients with PD were screened for eligibility and underwent baseline assessment but four dropped out before the program started. Eventually, 51 patients participated in the exercise program (Figure 1). Table 1 shows the baseline characteristics of the participants. The three groups were well matched regarding baseline characteristics, including gender [χ^2^(50) = 0.223, *p* = 0.894], age [*F*(2,50) = 0.502, *p* = 0.609], BMI [*F*(2,50) = 0.611, *p* = 0.547], PD years [*F*(2,50) = 0.739, *p* = 0.483], Hoehn and Yahr stages [χ^2^ (50) = 7.246, *p* = 0.510], use of levodopa [*F*(2,48) = 2.058, *p* = 0.139], and self-reported health [*F*(2,50) = 0.486, *p* = 0.618].

**TABLE 1:** Demographic information of participants among the Yang-ge dancing, conventional exercise, and conventional exercise plus music groups.

	CEM (*n* = 15)	CE (*n* = 19)	YG (*n* = 17)
Gender (*n*)			
Male	7	9	8
Female	8	10	9
Age (year)	67.18 (7.20)	69.16 (6.17)	67.40 (6.06)
BMI	23.21 (3.67)	23.41 (3.60)	24.50 (3.24)
PD years	5.03 (3.66)	4.89 (3.53)	3.65 (3.37)
H&Y stage (*n*)			
I	3	5	4
II	9	8	9
III	5	6	2
Levodopa (mg)	361 (155)	273 (100)	279 (168)
Health	2.88 (0.60)	3.05 (0.91)	3.13 (0.64)

YG, Yang-ge dancing; CE, conventional exercise; CEM, conventional exercise plus music; BMI, body mass index; PD years, years since diagnosed with Parkinson’s disease; H&Y stage, Hoehn & Yahr stage; Levodopa, current daily dose; Health, self-reported overall health status.

### Behavioral outcomes

To evaluate overall motor function changes by the varied exercise treatments, a 2 × 3 mixed MANOVA with repeated measures was conducted on the motor function using the four motor assessments (i.e., TUG, PPT, UPDRS—motor, and BBS) as dependent variables. The factors were time occasion (i.e., baseline and posttest) and group (i.e., YG, CE, and CEM). Descriptive data for the dependent variables are listed in Table 2. Preliminary data screening did not indicate serious violations of the basic assumptions. Two participants (one in the CE and one in the CEM) missed the PPT test as visual issues, so the mean imputation (i.e., replace the missing values by means, Warner, 2012) was applied. Examination of histograms and skewness/kurtosis ratios suggested that the four dependent variables were approximately normally distributed. Calculation of Z scores using the criterion of >±3.29Z as a potential outlier (Tabachnick and Fidell, 2007) indicated that no outlier exists in the dataset. As Table 1 shows, each group had 15–19 participants, suggesting approximately equal sample sizes across groups. Levene’s test yielded non-significant (*p*s > 0.05) difference in the variances of four outcome variables across the groups and time occasions, indicating the homogeneity of variance assumptions was not violated. The Box’s *M* test of equality of covariance matrices did not indicate a significant violation of homogeneity of covariance matrices across conditions, Box’s *M* = 80.695, *p* = 0.848. Levene’s test of equality of error variances did not indicate any violation of the homogeneity of variance neither, *F*s < 2.238, *p*s > 0.05. The Bartlett’s test of sphericity indicated the significant intercorrelations of the dependent variables, Approximate χ^2^(28) = 152.13, *p* = 0.001. Supplementary Table 3 shows the pooled within-cell correlations among the four outcome variables. The correlations ranged from 0.40 to 0.95, the three correlations with *r* > 0.900 were all repeated measures (i.e., BBS pre and posttest, *r* = 0.921; TUG pre and posttest, *r* = 0.913; UPDRS—motor scale pre and posttest, *r* = 0.948); all the other correlations ranged from 0.400 to 0.710, thus none of the correlations raised concerns about collinearity.

**TABLE 2:** Descriptive statistics for dependent variables.

Variables	YG (*n* = 17)		CE (*n* = 18)		CEM (*n* = 14)	
	*M*	*SD*	*M*	*SD*	*M*	*SD*
**BBS**						
Baseline	46.35	5.53	43.89	5.85	44.86	6.20
Posttest	49.94	3.70	47.44	4.22	48.07	4.55
**TUG**						
Baseline	14.24	4.74	13.42	4.70	13.26	3.83
Posttest	11.69	3.99	12.36	3.43	10.88	2.86
**PPT**						
Baseline	23.59	6.26	25.94	4.99	26.07	7.54
Posttest	27.53	7.39	25.78	6.33	27.80	5.93
**UPDRS—Motor**						
Baseline	22.41	11.65	21.33	10.46	21.79	9.19
Posttest	19.82	10.77	18.06	9.76	18.50	8.43

YG, Yang-ge dancing; CE, conventional exercise; CEM, conventional exercise plus music. TUG, timed up and go test; PPT, Purdue pegboard test; UPDRS, Unified Parkinson’s Disease Rating Scale; BBS, Berg balance scale.

For the overall MANOVA, a significant time × group interaction was detected (using α = 0.05 as the criterion), with Wilks’s λ = 0.687, approximate *F*(8,86) = 2.219, *p* = 0.034 (Supplementary Table 4). The corresponding η_*p*_^2^ effect size of 0.171 indicated a large effect for this interaction. The main effect of time occasion was also statistically significant, Wilks’s λ = 0.269, approximate *F*(4,43) = 29.276, *p* = 0.001, η_*p*_^2^ = 0.731. The main effect of group was not significant, Wilks’s λ = 0.872, approximate *F*(8,86) = 0.763, *p* = 0.636, η_*p*_^2^ = 0.066.

Because the interaction was statistically significant and accounted for a relatively large proportion of variance, 2 × 3 (time × group) mixed factorial ANOVAs were conducted as *post-hoc* tests to explore the nature of the interaction. Two of the four outcome variables, TUG and PPT, showed significant interactions, *F*(2,46) = 3.627, *p* = 0.034, η_*p*_^2^ = 0.136, and *F*(2,46) = 4.613, *p* = 0.015, η_*p*_^2^ = 0.167, respectively. The other two outcome variables, the UPDRS—motor and BBS were not significant, *F*(4,46) = 0.235, *p* = 0.795, η_*p*_^2^ = 0.010, and *F*(4,46) = 0.096, *p* = 0.909, η_*p*_^2^ = 0.004, respectively. Summary statistics for the factorial ANOVAs can be seen in Supplementary Table 5.

**FIGURE 2:**
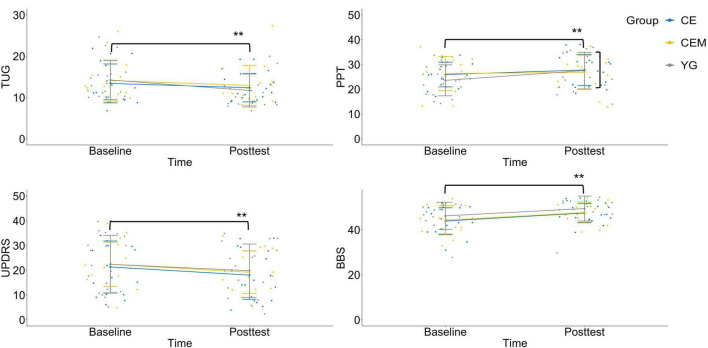
Interaction charts for behavioral outcomes. TUG, timed-up-and-go test; PPT, Purdue pegboard test; UPDRS, Unified Parkinson’s Disease Rating Scale-motor examination; BBS, Berg balance scale; CE, conventional exercise; CEM, conventional exercise plus music; YG, Yang-ge dancing. **p < 0.01.

For the TUG, the time × group interaction is depicted in Figure 2. Simple effects tests for time were then performed as follow up analysis. In comparison to the baseline assessment (Table 2; YG, *M* = 14.24, *SD* = 4.74; CE, *M* = 13.42, *SD* = 4.70; CEM, *M* = 13.26, *SD* = 3.83), all the three groups showed significantly less time to complete the TUG test at the posttest (YG, *M* = 11.69, *SD* = 3.99; CE, *M* = 12.36, *SD* = 3.43; CEM, *M* = 10.88, *SD* = 2.86), *t*(16) = 5.876, *p* = 0.001, *d* = 1.895, *t*(17) = 2.508, *p* = 0.016, *d* = 0.486, and *t*(13) = 4.990, *p* = 0.001, *d* = 1.411, respectively.

For the PPT, the time × group interaction is depicted in Figure 2. Simple effects tests for time were then performed as follow up analysis. The YG group showed significant improvement to the mean PPT score at the posttest (*M* = 27.53, *SD* = 7.39) than the baseline (*M* = 23.59, *SD* = 6.26), *t*(16) = 4.063, *p* = 0.001, *d* = 2.297. No statistical significance was observed in the CE group (Baseline, *M* = 25.94, *SD* = 4.99; Posttest, *M* = 25.78, *SD* = 6.33) and CEM group (Baseline, *M* = 26.07, *SD* = 7.54; Posttest, *M* = 27.80, *SD* = 5.93), *t*(17) = −0.177, *p* = 0.860, *d* = 0.048, and *t*(13) = 1.603, *p* = 0.116, *d* = 0.333, respectively.

For the UPDRS—motor examination, a graph of the group means across time occasions appears in Figure 2. The main effect was not statistically significant for group, *F*(2,46) = 0.091, *p* = 0.913, η_*p*_^2^ = 0.004; but was significant for time, *F*(2,46) = 39.677, *p* = 0.001, η_*p*_^2^ = 0.463. All the three groups demonstrated improved mean score on the UPDRS—motor scale in the posttest (YG, *M* = 19.82, *SD* = 2.37; CE, *M* = 18.06, *SD* = 2.31; CEM, *M* = 18.50, *SD* = 2.61) compared to baseline (YG, *M* = 22.41, *SD* = 2.56; CE, *M* = 21.33, *SD* = 2.49; CEM, *M* = 21.79, *SD* = 2.82), *t*(16) = 3.168, *p* = 0.003, *d* = 0.975, *t*(17) = 4.128, *p* = 0.001, *d* = 0.833, and *t*(13) = 3.647, *p* = 0.001, *d* = 1.558, respectively; whereas the differences among groups were not significant.

For the BBS, no significant interaction was detected as depicted in Figure 2. The main effect was not statistically significant for group, *F*(2,46) = 1.156, *p* = 0.324, η_*p*_^2^ = 0.048; but was significant for time, *F*(2,46) = 85.982, *p* = 0.001, η_*p*_^2^ = 0.651. All the three groups had improved mean BBS score in the posttest (YG, *M* = 49.94, *SD* = 3.70; CE, *M* = 47.44, *SD* = 4.22; CEM, *M* = 48.07, *SD* = 4.55) compared to baseline (YG, *M* = 46.35, *SD* = 5.33; CE, *M* = 43.89, *SD* = 5.85; CEM, *M* = 44.86, *SD* = 6.20), *t*(16) = 5.704, *p* = 0.001, *d* = 1.436, *t*(17) = 5.820, *p* = 0.001, *d* = 1.568, and *t*(13) = 4.638, *p* = 0.001, *d* = 1.039, respectively; whereas the differences among the groups were not significant.

The nature of the statistically significant time × group interaction can be summarized as follows. All three exercise prescriptions had significantly better average motor function than the baseline condition. However, of the three interventions, the CEM and YG showed greater improvement on the TUG test than the CE. The YG group showed better improvement on the PPT score than the other two interventions.

### Feasibility

In all, 100% of the YG (17 out of 17 participants), 100% of the CE (18 out of 18 participants), and 94% of the CEM (15 out of 16 participants) completed the trial. Adherence to the intervention was excellent with 99% course participation for the YG (335 out of 340 sessions were attended), 96% course participation for the CE (347 out of 360 sessions were attended), and 98% course participation for the CEM (275 out of 280 sessions were attended).

No major adverse events were noted during the intervention (Table 3). Over the 4 weeks of intervention, no falling occurred in any of the groups, 16 participants (5 in the YG, 6 in the CE, and 5 in the CEM) reported muscle soreness or pain, 2 participants reported dizziness or faintness (1 in the CE and 1 in the CEM), 1 participant in the YG reported symptoms of hypotension, 10 participants reported joint pain (4 in the YG, 2 in the CE, and 4 in the CEM), and 3 participants reported spasm (1 in the CE and 2 in the CEM).

**TABLE 3:** Adverse events among the yang-ge dancing, conventional exercise, and conventional exercise plus music groups.

	YG	CE	CEM
Falling	0	0	0
Muscle soreness or pain	5	6	5
Dizziness or faintness	0	1	1
Symptoms of hypotension	1	0	0
Joint pain	4	2	4
Spasm	0	1	2

YG, Yang-ge dancing; CE, conventional exercise; CEM, conventional exercise plus music.

## Discussion

This randomized controlled trial investigated the effects of exercise therapy and RAS and examined the feasibility of Yang-ge dancing for PD. The trial revealed three main findings. First of all, the groups that performed the exercise with music (i.e., the YG and CEM) had greater improvement in mobility, as compared with the group that did the exercise without music (i.e., the CE group). Secondly, the YG demonstrated greater improvements in manual dexterity compared to the other two groups. Thirdly, Yang-ge dancing is feasible and effective in enhancing motor function PD.

The findings of the study confirmed the effectiveness of participation in exercise (either conventional, or with/without music) for motor symptoms of PD, as well as in balance, which has been widely reported (Gobbi et al., 2009; Ni et al., 2018). The results also indicated that Yang-ge dancing has the potential to improve motor symptoms and balance performance in patients with PD as effectively as conventional PD exercise therapy. However, other exercise studies with such intervention duration and frequency (i.e., 5 sessions per week for 4 weeks) tended to demonstrate greater improvements in balance, which had about 6–10 point differences on the BBS following intervention (Bang and Shin, 2017; Palamara et al., 2017; Clerici et al., 2019) but only 3–4 points in the present study. The minor changes in the BBS were likely due in part to a ceiling effect. The patients included in present study were more within mild or moderate disease stages, which balance impairment has not yet been serious enough while under the influence of medication. Perhaps a measurement with higher precision (e.g., dynamic posturography) would be better to detect any changes in balance to patients within mild to moderate stages of PD.

The study provides evidence of additional benefits of exercise associated with RAS in improving functional mobility. This extra therapeutic effects can partly be explained by whole-body anticipatory and compensatory responses: The auditory stimuli increased attention and sensorimotor integration (i.e., the process whereby sensory input is integrated by the central nervous system; Abbruzzese and Berardelli, 2003), thus internal signals from the basal nucleus were stimulated to facilitate the execution of movements (dysfunction of the basal ganglia-motor cortex circuits is an accepted view on the pathophysiology of PD; Abbruzzese and Berardelli, 2003; Capato et al., 2020). Moreover, music may elicit emotional responses, as moving to music activates endorphin-related pleasure circuits in the brain, and the rhythm may promote that satisfactory patterning (Blood and Zatorre, 2001) which in turn may have enhanced the adherence and then ensured the efficacy of the intervention. The TUG combines walking, transferring, and turning, thereby mimicking everyday activities, which is also a demonstration of balance capacity on tasks of daily living with higher complexity. Such a finding provides further support for a stronger beneficial effect of exercise with music than exercise without music.

Participants in the YG group demonstrated a greater improvement in manual dexterity than those in the other groups, with an approximate 4-point increase in the PPT for the YG group, which is clinically meaningful. Previous exercise studies tended to report fewer effects on manual dexterity performance (Fernandez et al., 2015; Rios Romenets et al., 2015). It was commonly attributed to no specific hand movements being practiced with exercise therapy (Qutubuddin et al., 2013; van der Kolk et al., 2019). Previous researchers have demonstrated that manual dexterity can be enhanced through manipulative skill practice in patients with PD (Song et al., 2018; Fernández-González et al., 2019). As dancing with manipulating towels or hand fans is a significant characteristic of Yang-ge and throughout the intervention, the observed improvements in manual dexterity may be reflective of a global impact of exercise that involves manipulative skill practice and is consistent with the suggestion that participation in exercise may have disease-modifying effects, especially on bradykinesia (Duncan and Earhart, 2012).

As shown in the trial, Yang-ge dancing is a feasible exercise. Excellent adherence (99% course participation) was observed over the intervention. The high adherence, the zero drop-out rate, and the positive feedback to the intervention confirmed this observation. Participants found the Yang-ge dancing highly enjoyable and expressed overall satisfaction with the course, instructor, and care. The social interaction, social support, and social influences that emerged from the Yang-ge dancing also have a positive effect, which is with the potential to be highly motivational for PD patients to engage in physical activities in the long run.

Clinically, changes in motor symptoms, balance, mobility, and manual dexterity indicate increased potential for effectively performing daily life functions for the PD patients. Improvement in balance performance may increase functional ability in daily life, including transitioning between sitting and standing positions, turning, and walking, which are also the key components of functional mobility. Indirectly, these improvements may lead to a reduced risk of falls, which are a common life-threatening event in patients with PD (Pickering et al., 2007). The evidence of functional mobility also supports the efficacy of music as rhythmic auditory stimulation in alleviating motor symptoms associated with PD. Music can be integrated into PD exercise therapy to increase enjoyment of participating in therapy. Changes in manual dexterity may suggest the benefits of integrating movement exercises for the upper extremities and hands into PD exercise therapy.

The trial has potential limitations. First, the off-medication state of the patients was not evaluated, thus the effect of pharmacological treatment was not screened out. The patients had been required to not change their medication use over the experiment to minimize potential confounders. It would be clinically more helpful if the effect of medication could be partitioned out. Second, gender difference in the incidence and motor symptoms of PD had been reported in previous research (Solla et al., 2012; Dorsey et al., 2018), but was not examined in the present study due to the limited sample size. In the present study, female participants tended to be more motivated and positive toward participation than males. This was also reflected in the male-to-female ratio of the participants, as Dorsey et al. (2018) reported, the male-to-female PD ratio was about 1.40 globally, about 1.10 in China (Zou et al., 2015), but was 0.89 in the present study. The difference in sex ratio may have introduced biases in the results. Third, the limited improvement of manual dexterity in the CE and CEM groups might also be attributed to the fact that no manipulative-specific practice was included in the exercise protocols. Finally, the impact of non-exercise factors (e.g., the person-to-person interactions, music, and outdoor activity) may have mediated or moderated the improvements in motor function, so the net gain of exercise could not be gauged. Future research may consider enhancing the control of interventions, including the medical management, gender difference, and non-exercise factors. Additionally, biomechanical, physiological, and neuroimaging technologies should be applied to clarify and confirm the mechanisms involved.

## Conclusion

Yang-ge dancing appears to be feasible and effective as an alternative of exercise therapy designed to improve motor functions in patients with PD. The Yang-ge dancing program had the same benefits for improving PD motor symptoms and balance function as conventional PD exercise therapy. In respect to functional mobility, the Yang-ge dancing and conventional exercise with music were examined to be more effective than the conventional exercise without music, which may have partially examined the effects of rhythmic auditory stimulation. In addition, Yang-ge dancing demonstrated greater benefits in manual dexterity compared to conventional PD exercise therapy.

## Data availability statement

The raw data supporting the conclusions of this article will be made available by the authors, without undue reservation.

## Author contributions

FL carried out the experiment. MZ wrote the manuscript with support from ZL and DW. XB helped supervise the program. All authors contributed to the article and approved the submitted version.

## Supplementary material

Supplementary Video PD Yang-ge Dancing Video (Available online at: https://www.youtube.com/watch?v=4qBfwQgA72w).
