# Supplementary material for: The comparative effects of exercise type on motor function of patients with Parkinson’s disease: A three-arm randomized trial
Source: Front Hum Neurosci. 2022 Dec 1;16:1033289. doi: 10.3389/fnhum.2022.1033289 (PMC9751317; doi:10.3389/fnhum.2022.1033289)
Supplement: Supplementary file 1 [file Data_Sheet_1.PDF]

Supplementary Materials

Table S1. The Yang-ge Dancing Practice Tasks .... - 1 -

Table S2. Conventional Exercise Practice Complete Tasks .... - 2 -

Table S3. Intercorrelations of the Dependent Variables 4

Table S4. 2 × 3 Mixed Multivariate Analysis of Variance... 5

Table S5. Analysis of Simple Main Effects to Describe the Nature of Interaction 6

Table S1

The Yang-ge Dancing Practice Tasks

Practice Tasks
Seated toe point and kick
Seated leg lifting
Seated up-and-down leg march
Seated towels turning practice
Seated towels twisting practice
Kicking forward
Kicking back
Walking
Three steps walking and a stop
Step touch

Table S2

Conventional Exercise Practice Complete Tasks

Practice Tasks
Flexibility
Swan
Tib touches
Zipper stretch
Spinal twist
Calf stretch
Strength exercise
Chest press (with a 0.5kg water bottle on each hand)
Two-arm row (with a 0.5kg water bottle on each hand)
Overhead press (with a 0.5kg water bottle on each hand)
Hip abduction and adduction
Toe raises
Heel raises
Chair stands
Cardio exercise
Marching in place
Butterfly wings
Standing rowing
Balance
Standing (with eye closed)
Tandem standing (with eye closed)
Alternate weight shifts

Alternate heel lifts
Alternate toe touches to front/sides
Alternate heel touches to front/sides
Coordination
Marching with alternately swinging elbows
Marching with hand clapping
Leg lifting with cross touching

Table S3

Intercorrelations of the Dependent Variables

		Pre				Post			
		BBS	TUG	PPT	UPDRS	BBS	TUG	PPT	UPDRS
Pre	BBS	—	0.64	0.44	0.45	0.92	0.57	0.48	0.40
	TUG		—	0.59	0.71	0.59	0.91	0.60	0.67
	PPT			—	0.53	0.42	0.55	0.78	0.47
	UPDRS				—	0.43	0.65	0.61	0.95
Post	BBS					—	0.54	0.51	0.40
	TUG						—	0.59	0.65
	PPT							—	0.61
	UPDRS								—

Note. BBS, Berg balance scale; TUG, timed up and go test; PPT, Purdue pegboard test; UPDRS, Unified Parkinson's Disease Rating Scale - Motor section. All the correlations are significant at the 0.01 level (2-tailed).

Table S4

2 × 3 Mixed Multivariate Analysis of Variance Comparing the Effect of Exercise Treatment on Motor Functions across Two Test Occasions

	Wilk's λ	*F*	*df*	*p*	η_p^2
Between Subjects					
Group	0.872	0.763	8,86	0.636	0.066
Within Subjects					
Time	0.269	29.276	4,43	0.001	0.731
Time × Group	0.687	2.219	8,86	0.034	0.171

Note. df is presented as Hypothesis df, Error df.

Table S5

Analysis of Simple Main Effects to Describe the Nature of Group × Time Interaction: 2 × 3 Mixed Analyses of Variance Comparing the Effect of Exercise Treatment on Motor Functions across Two Test Occasions

	SS	*df*	*MS*	*F*	*p*	η_p^2
Group						
TUG	14.790	2	7.395	0.241	0.787	0.010
PPT	31.088	2	15.544	0.208	0.813	0.009
UPDRS	36.597	2	18.299	0.091	0.913	0.004
BBS	110.893	2	55.447	1.156	0.324	0.048
Time						
TUG	96.579	1	96.579	60.453	0.001	0.568
PPT	81.070	1	81.070	10.130	0.003	0.180
UPDRS	225.379	1	225.379	39.677	0.001	0.463
BBS	288.712	1	288.712	85.982	0.001	0.651
Time × Group						
TUG	11.587	2	5.794	3.627	0.034	0.136
PPT	73.830	2	36.915	4.613	0.015	0.167
UPDRS	2.666	2	1.333	0.235	0.792	0.010
BBS	0.642	2	0.321	0.096	0.909	0.004
Error (time)						
TUG	73.489	46	1.598			
PPT	368.149	46	8.003			
UPDRS	261.293	46	5.680			
BBS	154.460	46	3.358			
